# Personalization of electro-mechanical models of the pressure-overloaded left ventricle: fitting of Windkessel-type afterload models

**DOI:** 10.1098/rsta.2019.0342

**Published:** 2020-05-25

**Authors:** Laura Marx, Matthias A. F. Gsell, Armin Rund, Federica Caforio, Anton J. Prassl, Gabor Toth-Gayor, Titus Kuehne, Christoph M. Augustin, Gernot Plank

**Affiliations:** 1 Gottfried Schatz Research Center for Cell Signaling, Metabolism and Aging - Division of Biophysics, Medical University Graz, Graz, Austria; 2 Institute for Mathematics and Scientific Computing, University of Graz, Graz, Austria; 3 Department of Cardiology, Medical University Graz, Graz, Austria; 4 Institute for Cardiovascular Computer-assisted Medicine (ICM), Charité - Universitätsmedizin Berlin, Berlin, Germany; 5 Department of Imaging and Congenital Heart Disease, German Heart Center Berlin, Berlin, Germany

**Keywords:** aortic stenosis, coarctation, pressure gradient, heart failure

## Abstract

Computer models of left ventricular (LV) electro-mechanics (EM) show promise as a tool for assessing the impact of increased afterload upon LV performance. However, the identification of unique afterload model parameters and the personalization of EM LV models remains challenging due to significant clinical input uncertainties. Here, we personalized a virtual cohort of *N* = 17 EM LV models under pressure overload conditions. A global–local optimizer was developed to uniquely identify parameters of a three-element Windkessel (Wk3) afterload model. The sensitivity of Wk3 parameters to input uncertainty and of the EM LV model to Wk3 parameter uncertainty was analysed. The optimizer uniquely identified Wk3 parameters, and outputs of the personalized EM LV models showed close agreement with clinical data in all cases. Sensitivity analysis revealed a strong dependence of Wk3 parameters on input uncertainty. However, this had limited impact on outputs of EM LV models. A unique identification of Wk3 parameters from clinical data appears feasible, but it is sensitive to input uncertainty, thus depending on accurate invasive measurements. By contrast, the EM LV model outputs were less sensitive, with errors of less than 8.14% for input data errors of 10%, which is within the bounds of clinical data uncertainty.

This article is part of the theme issue ‘Uncertainty quantification in cardiac and cardiovascular modelling and simulation’.

## 1. Introduction

Pressure overload as induced by pathologies such as aortic valve stenosis (AS) or coarctation (CoA) imposes a significant increase in afterload upon the left ventricle (LV), which may impair myocardial energetics and drive maladaptive remodelling processes, eventually leading to heart failure (HF). Computer models of LV electro-mechanics (EM) show high promise as a clinical research tool for quantitatively assessing the impact of increased afterload upon LV function and, potentially, also for predicting acute and chronic outcomes of interventions such as aortic valve repair/replacement or the stenting of a CoA. Such advanced diagnostic applications are critically dependent on the ability of models to accurately represent loading conditions for a given patient. However, the choice of an appropriate model representing LV afterload and the accurate identification of unique sets of model parameters are non-trivial.

First of all, there is no consensus on the choice of an afterload model that would best represent circulatory impedance, which indicates *structural uncertainty*. A number of models have been reported in the literature, ranging from simpler lumped zero-dimensional (0D) Windkessel-type models comprising two, three or four elements [1–12] which account for resistive and reservoir effects, to more advanced one-dimensional (1D) models derived from the Navier–Stokes equations [13–17] which also consider pulse wave transmission effects. 1D models are preferred over 0D models when distributed properties and their impact upon central pressure waveforms and associated markers such as pulse wave velocity are under investigation. 0D models are thus unable to account for effects such as pressure wave augmentation which render the estimation of central aortic pressure governing LV afterload from cuff measurements a challenging endeavour [18–20]. In general though, as a model of global LV afterload, 0D models have been preferred as their lower number of parameters is more likely identifiable with data typically available in the clinic.

Beyond the structural uncertainty linked to the choice of a specific afterload model, key parameters characterizing circulatory impedance (i.e. LV pressure, *p*_lv_, central aortic pressure, *p*, the pressure drop across the aortic valve, Δ*p*_av_ = *p*_lv_ − *p*, and aortic flow, *q*) show beat-to-beat variability and their measurements are afflicted with significant errors, introducing *residual* and *observational uncertainties*, respectively. These uncertainties are exacerbated in clinical scenarios where the catheterization of patients is avoided. In these cases, invasive pressure recordings are not available, necessitating the indirect inference of aortic pressure from cuff measurements of brachial pressures and the calibration of trans-valvular pressure drops from flow measurements which are, due to a number of simplifying assumptions, inherently inaccurate [21]. Beyond uncertainties related to the afterload, combined EM LV models are further affected by *geometric uncertainties* due to image resolution, segmentation and mesh fitting, *condition uncertainties* due to initial and boundary conditions (BCs) as well as *complex input uncertainties* due to limited knowledge of complex spatially heterogeneous factors such as the electrical activation sequence driving contraction, fibre and sheet arrangements, heterogeneity in ion channel expression or sarcomere dynamics. Finally, also *simulator uncertainty* due to numerical approximation errors may play a role, but may be, relative to other more significant uncertainties, of lesser relevance. Finally, even in the absence of any measurement errors, a unique identification of model parameters may not be feasible on mathematical grounds, as more than one choice of model parameters may yield the same goodness of fit to the measured data. All these factors combined contribute to the *input uncertainty* of an EM LV model.

Considering the significant input uncertainty in EM LV models raises the question as to which degree these propagate and affect the simulated model outputs. In this study, we sought to quantitatively relate the influence of input uncertainty upon afterload model parameter estimation and, in turn, the arising model parameter uncertainty upon *output uncertainty* of the combined EM LV model. For this sake *N* = 17 personalized finite-element (FE) EM LV models coupled to a three-element Windkessel (Wk3) model representing afterload were built from clinical data of patients treated for AS (*N*_AS_ = 10 cases) or CoA (*N*_CoA_ = 7 cases). An automated global–local optimization method was developed for identifying parameters of the Wk3 model of afterload both with and without invasive pressure recordings available in the CoA and AS cases, respectively. To yield reproducibly unique solutions, clinical data were pre-processed to mitigate artefacts and achieve consistency across data sources by, for example, synchronizing pressure and flow traces and adjusting for differences due to altered heart rate, and physiological box constraints were imposed to limit the search space.

For all cases under study, our optimization approach identified a globally optimal set of Wk3 parameters under the given physiological constraints. Using a minimum number of inputs, parameters were found automatically without any operator interventions within less than 1 min. Simulation results obtained with the combined EM LV models showed close agreement, within the limits of clinical data uncertainty, with all available measurements in all *N* = 17 cases. Sensitivity analysis revealed a significant dependence of estimated Wk3 parameters on observational uncertainty. However, predictions of the EM LV models were highly robust with regard to uncertainty of afterload model parameters. Even when considering large errors in input data of 10%, model outputs remained within an envelope of less than 8.14% deviation, which is well within the bounds of clinical data uncertainty.

## 2. Methods

### (a) Patient data

In a prospective clinical study, patients were identified who met the inclusion criteria for the aortic CoA arm or the aortic valve disease (AVD) arm of the *CARDIOPROOF* trial (NCT02591940). From this study, data of *N*_AS_ = 10 AVD patients suffering from AS and *N*_CoA_ = 7 CoA patients with clinical indication for aortic valve treatment and stenting of CoA were selected. AS treatment indicators included valve area and/or systolic pressure drop across the valve. CoA treatment indicators included an echocardiographic measured peak systolic pressure gradient across the stenotic region of greater than 20 mmHg (2.66 kPa) and/or arterial hypertension. The study was approved by the institutional Research Ethics Committee following the ethical guidelines of the 1975 Declaration of Helsinki. Written informed consent was obtained from the participants’ guardians.

Details on clinical protocols and acquisition of data used in this study have been reported in detail elsewhere [22]. Acquired clinical data include anatomical 3D-whole-heart (3DWH) magnetic resonance imaging (MRI) scans, LV volume traces, *V*_lv,m_(*t*), as derived from short-axis (SAX) cine MRI scans, image-based ultrasound (US-echo) estimations of pressure drop across the valve and/or coarctation at peak flow, Δ*p*_av,m_, and cuff pressure measurements yielding diastolic and systolic brachial pressures, *p*_dia,cuff_ and *p*_sys,cuff_. In addition, in all CoA cases, invasively recorded pressures in LV, *p*_lv,m_(*t*), and aorta, *p*_m_(*t*), were also available.

### (b) Model-fitting procedures

**Figure 1.**
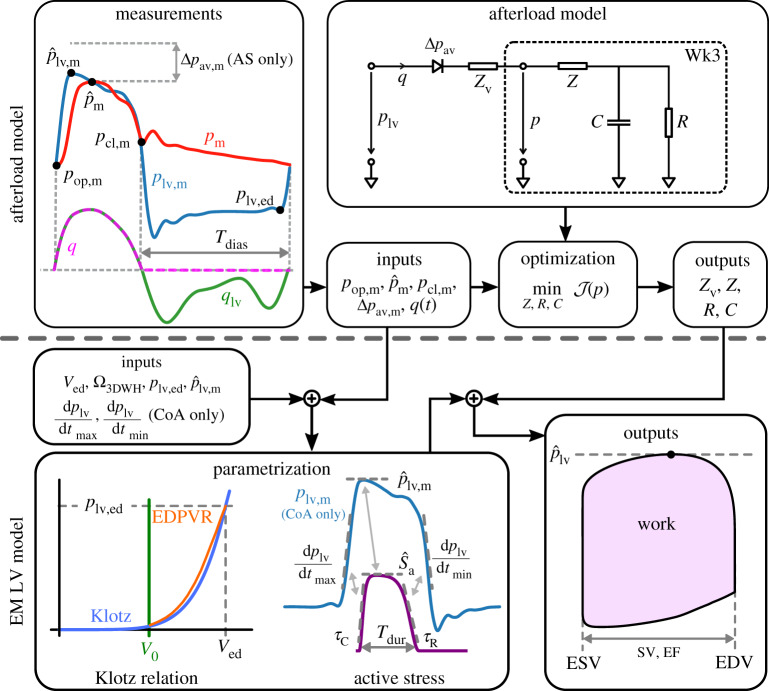
Overview of model workflow for fitting afterload model (upper panel) and combined EM LV model (lower panel). In a first step, the Wk3 afterload parameters *Z*, *Z*_v_, *R* and *C* were identified using measured haemodynamic parameters *p*_op_, m, ${\hat{p}}_{\text{m}}$, *p*_cl_, m, Δ*p*_av_, m and *q*(*t*) = −d*V*/d*t* as inputs. For AS cases, *p*_op_, m and ${\hat{p}}_{\text{m}}$ were estimated from cuff measurements, whereas *p*_cl_, m was estimated from empirical reference data. Subsequently, the EM LV model is fitted. First, the biomechanical bulk modulus *C*_Guc_ is adjusted to fit the passive behaviour of the LV model to the empirical approximation of the end-diastolic pressure–volume relation (EDPVR) due to Klotz, using {*V*_ed_, *p*_lv_, ed} as inputs. For AS cases *p*_lv_, ed was estimated from empirical reference data. Using the fitted afterload model coupled to the EM LV model through a resistive valve model, the active stress model is parametrized using fixed-point iterations to adjust the phenomenological active stress model parameters, $\left\{\tau_{\text{C}},{\hat{S}}_{\text{a}},T_{\text{dur}},\tau_{\text{R}}\right\}$, using the discrepancy between measured and simulated p−V metrics during isovolumetric contraction and ejection. In the diagram: EF, ejection fraction; ESV, end-systolic volume; and EDV, end-diastolic volume. (Online version in colour.)

Measured pressure and volume data were pre-processed to mitigate data inconsistencies (see electronic supplementary material, §1(a) for details). Pre-processed data were used then for fitting the EM LV model in a two-step procedure, where first the afterload model parameters were identified from haemodynamic measurements only and, subsequently, parameters of the integrated EM LV model components representing passive biomechanical behaviour and active stress generation were identified. An overview of the overall workflow is given in figure 1.

#### (i) Identification of afterload model parameters

The afterload imposed by the arterial system on the LV is represented by a Wk3 model given as

2.1 $$\frac{\text{d}p}{\text{d}t}=\left(\frac{1}{C}+\frac{Z}{RC}\right)q+Z\frac{\text{d}q}{\text{d}t}-\frac{1}{RC}p,$$

where *q* is the flow across the aortic valve in ml ms^−1^, *t* is the time in ms, *p* is the pressure in the aorta ascendens in kPa, *R* and *C* are total arterial resistance and compliance in kPa ms ml^−1^ and ml kPa^−1^, respectively, and *Z* is the characteristic aortic impedance in kPa ms ml^−1^ [23].

Rendering the Wk3 model patient-specific requires the identification of parameters *Z*, *R* and *C* from measurements of *p*(*t*) and *q*(*t*). For a given set of parameters {*Z*, *R*, *C*}, one can use *p* as given and solve (2.1) for *q*, or vice versa. While the temporal resolution of *q*(*t*) as measured by cine MRI is lower than that of *p*(*t*), because *q*(*t*) can be measured non-invasively and the temporal resolution, of the order of some tens of milliseconds, suffices to resolve the dynamics with sufficient accuracy, we treat *q* as given and seek to find the set {*Z*, *R*, *C*} that minimizes the difference between measured pressure, *p*_m_, and simulated pressure, *p*. Furthermore, to make the parametrization procedure as generally applicable as possible, we refrain from using *p*_m_(*t*) and rely on a set of characteristic data points extracted from *p*_m_(*t*). As *p*_m_(*t*) requires invasive catheterization, these data may or may not be available depending on the specific clinical procedures (see §2a). For instance, in the data of the *CARDIOPROOF* cohort, invasive pressure measurements were not available for AS cases. Thus, we use only the opening and closing pressure of the aortic valve, *p*_op_, m and *p*_cl_, m, respectively, and peak pressure, ${\hat{p}}_{\text{m}}$, as pointwise measures to gauge the goodness of fit. In the absence of a measured time trace *p*_m_(*t*), these values must be estimated from cuff measurements, a procedure afflicted with significant uncertainties [18] and empirical reference data (see electronic supplementary material, §2(a)). In all AS cases, LV peak systolic pressure was estimated as ${\hat{p}}_{\text{lv, est}}=p_{\text{sys, cuff}}+\Delta p_{\text{av, m}}$, where the peak pressure drop Δ*p*_av_ was estimated from US flow measurements, based on Bernoulli’s principle [21, eqn. 1]. The impedance of the resistive diode used to couple the Wk3 afterload model to the EM LV model was estimated as

2.2 $$Z_{\text{v}}\approx \frac{\Delta p_{\text{av}}}{\hat{q}},$$

where *Z*_v_ represents the additional impedance of the aortic valve due to the presence of AS.

Wk3 model parameters *Z*, *R* and *C* were fitted by minimizing the cost functional

2.3 $$J(p)=\frac{\omega_{0}}{2}{\left({\hat{p}}_{\text{m}}-\hat{p}\right)}^{2}+\frac{\omega_{1}}{2}{\left(p_{\text{op, m}}-p_{\text{ed}}\right)}^{2}+\frac{\omega_{2}}{2}{\left(p_{\text{cl, m}}-p_{\text{cl}}\right)}^{2}.$$

Therein, the difference between measured and simulated pressures *p*_m_ and *p* was evaluated in terms of peak pressure, ${\hat{p}}_{\text{m}}$ and $\hat{p}$, valve opening versus end-diastolic pressure, *p*_op,*m*_ and *p*_ed_, and valve closing pressure, *p*_cl_, m and *p*_cl_ (see figure 1, upper left panel). Values of *ω*_*i*_ result from weighting factors *γ*_*i*_, which were chosen so that each term of the cost functional contributes to the same extent to the overall cost divided by the respective squared mean values obtained from cohort data (see electronic supplementary material, §1(c)).

The minimization was performed subject to the Wk3 model (2.1) with *p*(0) = *p*_op_, m and measured *q*_m_. Five additional inequalities were prescribed:

2.4 $$1\leq Z,\;1\leq C\leq R,\;b_{0}\leq \frac{RC}{T_{\text{dias}}}\leq b_{1},\;b_{2}\leq \frac{p_{\text{cl}}}{\text{MAP}},\;b_{3}\leq p_{\text{cl}},$$

where the parameters *b*_*i*_ ensure that quantities derived from *p* such as mean arterial pressure (MAP) or *p*_cl_ do not exceed their physiological ranges; and *T*_dias_ denotes duration of diastole (figure 1). The inequalities were included by penalty methods using suitably scaled max-functions. Exact values for *b*_*i*_ and a detailed description of the optimization method used can be found in the electronic supplementary material, §1(c) and §1(e), respectively.

#### (ii) Parametrization of the combined EM LV model

FE meshes of LV anatomy and aortic root were generated using established workflows described elsewhere [24,25]. Parametrization of the LV model followed closely a previously used protocol [26], but with an improved representation of mechanical BCs which better reflect the *in vivo* situation. Briefly, at the rim of the clipped aorta and over the epicardial surface, spring BCs were applied, which penalized displacement, either in any direction, at the aorta, or only along a direction normal to the epicardial surface in end-diastolic configuration. These normal spring BCs, implemented similarly to [27], mimic the effect of the pericardium which restricts changes of the outer shape of the heart. To match long-axis shortening of the LV during ejection between model and image-based kinematics, the spring constant was scaled gradually, changing from zero at the base to one at the apex. Moreover, to avoid a non-physiological rotation of the LV [28,29] due to the absence of the right ventricle in the model, additional spring BCs were applied at the right ventricular surface of the septum (see electronic supplementary material, §1(d)iii). Following [30], passive biomechanical parameters of the Guccione model [31] were determined by fitting the LV model to an empirical Klotz relation [32] (see electronic supplementary material, §2(c)ii). The unloaded EM LV model was inflated to *p*_lv_, ed and electrically activated to initiate mechanical contraction using a phenomenological length-dependent contractile model of active stress generation [33,34], and coupled to the patient-specific fitted afterload model using a valve represented as a resistive diode of resistance *Z*_v_. The coupled EM LV model was solved to compute LV pressure *p*_lv_, aortic pressure *p* and LV volume *V*_lv_ during isovolumetric contraction and ejection phase. Differences between computed and measured pressure and flow-based metrics were minimized by iterative adjustment of active stress model parameters using a fixed-point iteration (see electronic supplementary material, §2(c)iii).

### (c) Sensitivity analysis

Considering the significant observational and residual uncertainty of the input data, sensitivity analysis was performed to investigate the robustness of the implemented model fitting following a two-step procedure. First, the effect of uncertainties of input data comprising opening and closing pressure of the aortic valve, *p*_op,*m*_ and *p*_cl,*m*_, as well as aortic peak pressure, ${\hat{p}}_{\text{m}}$, upon the fitting of the afterload model was quantified, that is, $\left\{Z,R,C\}=f(p_{\text{op},m},{\hat{p}}_{\text{m}},p_{\text{cl},m}\right)$. Secondly, the impact of afterload model uncertainty upon the combined EM LV model was studied, that is, $\left\{\text{SV},\text{SW},t_{\hat{p}}\}=f(Z\pm \Delta Z,R\pm \Delta R,C\pm \Delta C\right)$, where SV, SW and $t_{\hat{p}}$ are stroke volume, stroke work and time occurrence of aortic peak pressure, respectively. Other measured input data such as LV volume *V*_lv_, m(*t*) and the derived aortic flow *q*(*t*) are also afflicted with uncertainties, but these remained unconsidered in the sensitivity analysis. EM model parameters (table 1) were fitted once for the initial input parameters and were subsequently held constant for the cases with input parameter perturbation.

**Table 1.** Fitted model parameters and goodness of fit for EM simulations of cases 10-AS and 02-CoA for the initial input parameters.

	fitted parameters					goodness of fit			
case ID	*C*_Guc_	${\hat{S}}_{a}$ (kPa)	*T*_dur_ (ms)	*τ*_C_ (ms)	*τ*_R_ (ms)	Δ_EDV_ (ml)	Δ_ESV_ (ml)	Δ_EF_ (%)	$\Delta_{\hat{\text{p}}}$ (kPa)
10-AS	0.4	95	495	70	70	0.40	10.39	5.07	0.25
02-CoA	0.8	57	575	105	90	0.08	0.40	0.16	0.32

Sensitivity analysis was performed using two distinct approaches, by independent variation (IV) and by combined variation (CV) of parameters. In the IV case vectors of size *N* = 100 were created for each input *p*_op_, m, ${\hat{p}}_{\text{m}}$ and *p*_cl_, m by even sampling a range of ±10% around the measured value. The Wk3 model-fitting algorithm was executed then varying only one input parameter at a time while keeping all others fixed. The impact upon EM LV was studied by using all possible *N* = 6 combinations of the extreme Wk3 parameters found and quantifying the deviation in model predictions $\left\{\Delta \text{SV},\Delta \text{SW},\Delta t_{\hat{p}}\right\}$ relative to the reference EM simulations where the mean parameters were used. Eight further simulations with combined input parameter variations were studied and added (see electronic supplementary material, §2(d)).

Alternatively, Saltelli’s extension of the Sobol sequence was used to generate *N* = 5000 samples of the input parameter space using the same range of ±10% around the measured value. An independent multivariate normal distribution was assumed with mean values *p*_op_, m, ${\hat{p}}_{\text{m}}$, *p*_cl_, m and variance *σ*^2^ = 0.2 kPa^2^. For all *N* = 5000 input parameter sets, Wk3 model parameters were fitted and their probability densities determined. Using the open Python library SALib V. 1.3.8 [35], first-order Sobol indices [36] for each output parameter were then computed for each input parameter, which reflect the contribution of each input parameter to the output variance.

### (d) Numerical solution

Spatio-temporal discretization of all PDEs and the solution of the arising systems of equations relied upon the Cardiac Arrhythmia Research Package (CARP) [37]. Numerical details on FE discretization as well as numerical solution of electrophysiology [38–40] and electro-mechanics [41] equations have been described in detail previously. Both electrophysiology and mechanics solver components have been validated in *N*-version benchmark studies [42,43]. Global and local optimization algorithms were implemented in Python V. 2.7.15 using NumPy V. 1.16.5 and SciPy V. 1.2.2.

## 3. Results

### (a) Fitting of afterload and EM LV model

**Figure 2.**
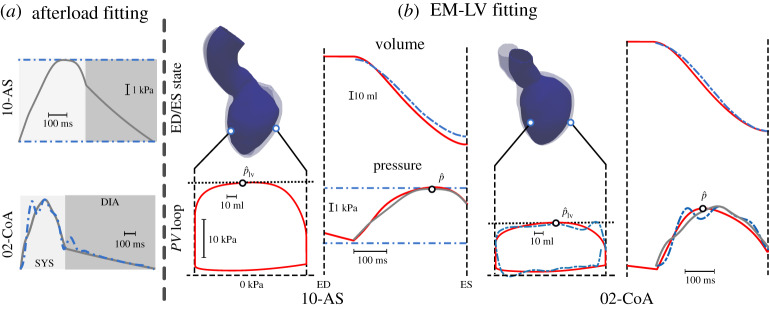
Results of (*a*) afterload fitting and (*b*) EM LV fitting for cases 10-AS and 02-CoA. (*a*) Fitted and measured pressure traces are drawn as solid grey and dashed-dotted blue traces, respectively. In case 10-AS only discrete pressure values were available (dashed-dotted blue). (*b*) *PV* loops simulated with the EM LV are shown over a full cardiac cycle, whereas the resulting LV volume and aortic pressure traces are shown only during ejection. Traces refer to EM LV simulation (solid red), measurements (blue dashed-dotted) and results of the afterload fitting, computed by solving the Wk3 equation (2.1). Gap in IVC phase of measured *PV* loop for the 02-CoA case can be explained by the low sampling resolution causing *V*_lv_, m to be not completely periodic. End-diastolic (ED, transparent) and end systolic (ES, solid blue) configurations of the LV are also shown. (Online version in colour.)

For all AS and CoA cases under study, Wk3 model parameters {*Z*, *R*, *C*} were identified by solving the optimization problem given in equations (2.3) and (2.4), using *p*_op_, m, ${\hat{p}}_{\text{m}}$, *p*_cl_, m and *q*(*t*), as derived from measurements in a pre-processing procedure (see electronic supplementary material, §1(a)), as inputs. The goodness of fit is shown for two representative cases, one AS (10-AS) and one CoA (02-CoA) case, in figure 2*a*. Afterload fitting results are summarized in table 2.

**Table 2.** Results of Wk3 fitting procedure for two representative cases 10-AS and 02-CoA.

case ID	*p*_op_ (kPa)	*p*_cl_ (kPa)	$\hat{p}$ (kPa)	*Z* (kPa ms ml^−1^)	*R* (kPa ms ml^−1^)	*C* (ml kPa^−1^)	RC (ms)
10-AS	10.00	15.45	17.87	9.26	97.46	8.42	820.60
02-CoA	6.54	8.52	13.34	12.75	80.40	38.66	3108.14

To ascertain that the minimum found is global within the physiologically plausible corridor imposed by the box constraints given in (2.4), the fitting procedure was repeated, using 100 randomly chosen initial guesses. In all cases, exactly the same minimizers were found, suggesting that the identified minima are indeed global and, thus, Wk3 parameters can be uniquely identified under the given constraints.

For all cases, anatomical models were built from images (electronic supplementary material, figure S4). Using the identified afterload model parameters, passive biomechanical and active stress parameters were identified in the combined EM LV model. Goodness of fit is illustrated in figure 2*b*, whereas identified parameters are given in table 1. For a complete list of fitted afterload and EM LV model parameters of all cases studied, we refer to electronic supplementary material, tables S3 and S4, respectively.

### (b) Sensitivity analysis

Sensitivity was analysed in a two-step procedure, where the sensitivity of the afterload model parameters to measurement errors was quantified first and then, subsequently, the sensitivity of biomechanical and haemodynamic model outputs of the EM LV model to afterload parameter uncertainties was studied.

Sensitivity of fitting {*Z*, *R*, *C*} to uncertainty in input data $\left\{p_{\text{op, m}},{\hat{p}}_{\text{m}},p_{\text{cl, m}}\right\}$ was analysed for all *N* = 17 cases by independently varying the input variables within a ±10% range. Relative sensitivities averaged over all cases are summarized in table 3.

**Table 3.** Maximum ± relative deviation of Wk3 parameters over all cases as a function of errors in the input data (for pathology specific results see electronic supplementary material, table S5), *p*_op,m_, ${\hat{p}}_{\text{m}}$ and *p*_op,cl_, and their relative importance for Wk3 fitting accuracy as measured by first-order Sobol indices {*S*^Z^, *S*^R^, *S*^C^} for each output *Z*, *R* and *C* in the cases 10-AS and 02-CoA.

					case 10-AS			case 02-CoA		
input varied	dev.	$Z_{\Delta \pm ,\text{max}}$ (%)	$R_{\Delta \pm ,\text{max}}$ (%)	$C_{\Delta \pm ,\text{max}}$ (%)	*S*^Z^	*S*^R^	*S*^C^	*S*^Z^	*S*^R^	*S*^C^
*p*_op,m_	±10%	+14.01/ − 28.53	+7.67/ − 5.67	+29.97/ − 23.99	0.000	0.278	0.305	0.060	0.636	0.299
${\hat{p}}_{\text{m}}$	±10%	+79.82/ − 87.01	+0.18/ − 0.88	+4.39/ − 0.52	0.566	0.010	0.003	0.845	0.000	0.000
*p*_cl,m_	±10%	+59.67/ − 85.35	+6.51/ − 6.51	+44.48/ − 29.37	0.417	0.695	0.659	0.089	0.358	0.669

Sensitivities of individual Wk3 parameters varied markedly. Overall, *R* was affected the least, with *R*_Δ±,max_ < 10%, i.e. a sensitivity <1 attenuated errors in input data. By contrast, *C*_Δ±,max_ and *Z*_Δ±,max_ both showed higher sensitivity, with relative errors ≫ 10%, that is, sensitivities of ≫ 1 led to a significant amplification of input data errors.

**Figure 3.**
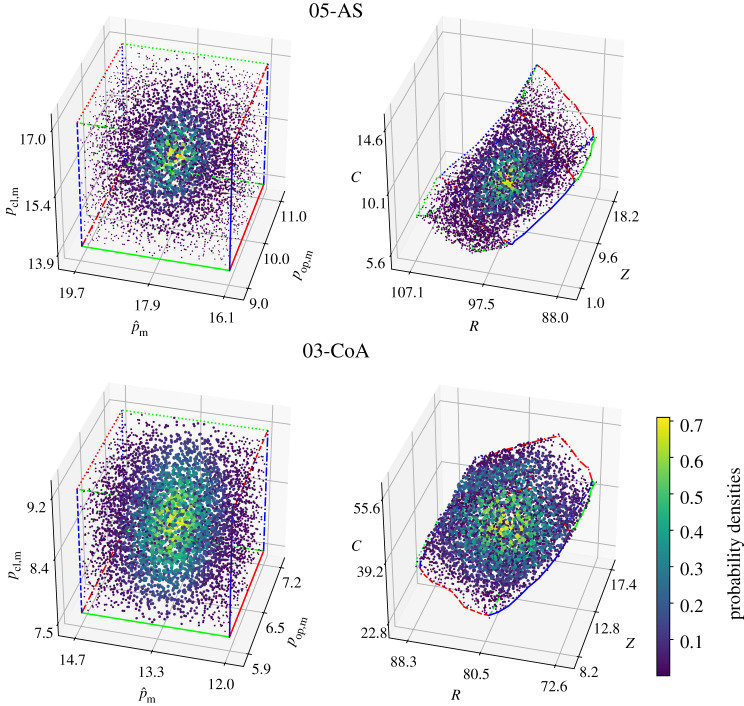
Input parameters $\left\{p_{\text{op, m}},{\hat{p}}_{\text{m}},p_{\text{cl, m}}\right\}$ and associated Wk3 parameters {*Z*, *R*, *C*} for cases 10-AS and 02-CoA. Distributions suggest a continuous mapping. An independent multivariate normal distribution with variance *σ*^2^ = 0.2 kPa^2^ was used to colour the samples. (Online version in colour.)

In addition, for the cases 10-AS and 02-CoA, the combined variation of input parameters based on a Sobol sampling was carried out. Qualitatively, the probability densities shown in figure 3 suggest a continuous mapping between input and output parameters, that is, small bounded measurement errors in the input data translate into small bounded errors in the output. Quantitatively, the higher Sobol indices indicate that errors in measuring the corresponding pressure input parameters carry more weight with regard to the variance of the output Wk3 parameters than those pressure measurements with a lower Sobol index (see table 3, rightmost columns).

**Figure 4.**
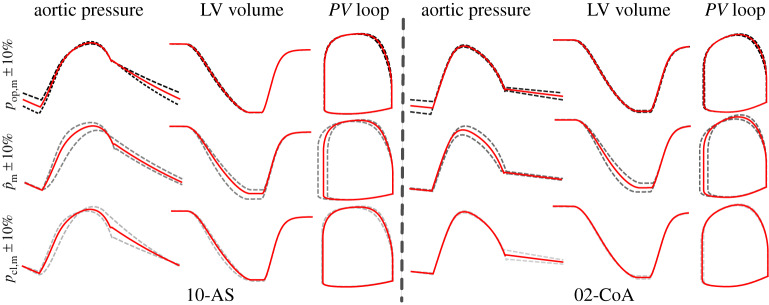
Effect of Wk3 parameter variability on physiological signal output of EM LV model. Pressure, volume and *PV* loops resulting from EM simulations with varied input parameters are shown for case 10-AS and case 02-CoA. Deviations due to changes in *p*_op,m_ (dashed black), ${\hat{p}}_{\text{m}}$ (dashed grey) and *p*_cl,m_ (dashed light grey) are visualized along with initial results in solid red. (Online version in colour.)

In a second study, the sensitivity of the EM LV model biomechanical and haemodynamic outputs, leaving all EM LV model parameters unaltered as fitted, was probed by perturbing Wk3 parameters within the range of values as induced by ±10% input data errors. Owing to the significant computational costs involved, the analysis was carried out only for the cases 10-AS and 02-CoA. The physiological envelope of Wk3 parameter uncertainty is illustrated in figure 4. Quantitative metrics are summarized in table 4. Results from eight further simulations with combined input parameter variations can be found in electronic supplementary material, table S6.

**Table 4.** Relative deviation of results of EM simulations from initial fit for the cases 10-AS and 02-CoA using six different Windkessel parameter sets as input, while keeping EM model parameters constant.

		case 10-AS			case 02-CoA		
	deviation	SV (ml)	SW (J)	$t_{\hat{p}}$ (ms)	SV (ml)	SW (J)	$t_{\hat{p}}$ (ms)
	0%	110.77	2.58	345	115.31	1.44	280

input varied		ΔSV (%)	ΔSW (%)	$\Delta t_{\hat{p}}$ (%)	ΔSV (%)	ΔSW (%)	$\Delta t_{\hat{p}}$ (%)
*p*_op,m_	−10%	0.09	−0.93	−0.29	1.27	−0.35	−0.36
*p*_op,m_	+10%	0.02	0.93	0.29	−1.18	0.14	0.00
${\hat{p}}_{\text{m}}$	−10%	8.14	6.00	6.67	6.52	3.62	−3.21
${\hat{p}}_{\text{m}}$	+10%	−5.76	−4.92	−2.61	−6.60	−4.74	2.50
*p*_cl,m_	−10%	1.99	2.36	−4.06	1.51	1.74	0.00
*p*_cl,m_	+10%	0.40	−1.08	4.93	−2.04	−2.44	0.00

Despite the marked variability in Wk3 parameters as induced by the 10% errors in input data (table 1), outputs of the EM LV model were affected rather moderately; only errors in ${\hat{p}}_{\text{m}}$ led to more notable changes.

## 4. Discussion

In this study, we describe a workflow for building EM LV models and their patient-specific parametrization. A series of pre-processing steps was implemented to mitigate artefacts introduced by input data uncertainty and to improve consistency between data acquired non-simultaneously under markedly different conditions. A particular focus was on identifying Wk3 afterload parameters from haemodynamic measurements. The parametrization workflow was evaluated under two clinical scenarios in the presence (in the CoA cases) and absence (in AS cases) of invasive pressure measurements, as the ability to parametrize EM LV models strictly non-invasively was deemed a key advantage in view of future clinical applications. Thus, the Wk3 parameter identification procedures were implemented to accommodate both scenarios, using only the point estimates of $\left\{p_{\text{op, m}},{\hat{p}}_{\text{m}},p_{\text{cl, m}}\right\}$ as input, but no pressure traces. As the optimization problem to be solved is non-convex, local optimization methods may get trapped in non-competitive local minima. Therefore, a novel combined global–local optimizer was developed. Using physiologically motivated box constraints, this optimizer finds global minima and, thus, facilitates the unique identification of Wk3 parameters. The method has proven robustness as testing 100 randomly chosen initial guesses yielded exactly the same parameter sets.

Sensitivity analysis was employed to quantify the dependence of estimated Wk3 parameters on input data uncertainty, revealing that *Z* is affected most notably. These errors in afterload model parameters also had an impact on the output of the EM LV model, but to a much lesser extent. Even when using the most extreme Wk3 parameters, the output of the EM LV model was only marginally altered, with variations well below the clinical observational uncertainty.

### (a) Fitting of global EM LV model

The global EM LV model was parametrized by fitting first the individual components independently. The constitutive biomechanical model was fitted to a Klotz relation, using *p*_ed_ and *V*_ed_ as input, the Wk3 parameters were fitted using *q*, *p*_op_, m, ${\hat{p}}_{\text{m}}$, *p*_cl_, m as inputs, and, finally, in the AS cases, the resistance *Z*_v_ was derived from estimates of the trans-valvular pressure gradient.

In a final step the global model was parametrized by identifying parameters of the active contractile model using the same input data as previously for fitting the Wk3 model. All assembled EM LV models fitted the data with sufficiently high accuracy, clearly within the range of input data uncertainty of the clinical measurements. In a few cases, the goodness of fit achieved in terms of SV was less accurate, with deviations ΔSV > 5%. Reasons were multifactorial. In the case of very high ejection fractions of >70%, discrepancies are likely to be due to limitations of the P1 and P0 FE types used. These are computationally efficient, but showed locking phenomena under the large deformations at such high ejection fraction (EF). Furthermore, the fixed-point iteration used treated each active stress model parameter independently and, owing to the costs of fitting the global EM LV system, the iteration was not repeated until a convergence criterion was met; rather, only one iteration step was executed.

### (b) Influence of uncertainties on Wk3 parameter estimation

Wk3 parameters were affected by input uncertainty to varying degrees. *R* representing the total arterial resistance was affected the least, with sensitivities <1 to all input parameters. This is plausible as *R* ≈ MAP/CO holds and both MAP and cardiac output (CO) are only very moderately affected by smaller changes of $\left\{p_{\text{op, m}},{\hat{p}}_{\text{m}},p_{\text{cl, m}}\right\}$ due to their integral nature. Similarly, since the compliance *C* of the arterial system governs the time constant *τ* = *R C* of the exponential decay in aortic pressure during diastole, a stronger dependence on *p*_op_, m and *p*_cl_, m and a weak dependence on ${\hat{p}}_{\text{m}}$ occurring during systole is expected. Finally, *Z* showed the highest sensitivity to input uncertainty. As any effects due to *Z* appear during systole only, its estimation depends therefore mostly on ${\hat{p}}_{\text{m}}$ and *p*_cl_, m. As shown in table 3 and electronic supplementary material, table S5, the sensitivity of *Z* to these inputs was very high. A more detailed investigation showed that the influence of *p*_cl_, m on *Z* correlates to the ratio $\left(p_{\text{cl, m}}-p_{\text{op, m}})/({\hat{p}}_{\text{m}}-p_{\text{op, m}}\right)$. The higher the ratio of the original input pressures, the more influence *p*_cl_, m had on *Z*; see electronic supplementary material, figure S5.

In view of our observations, a strictly non-invasive parametrization approach appears challenging to achieve if highly accurate estimation of Wk3 parameters is sought. While *p*_op_, m, in general, can be estimated reliably from cuff measurements, this is not the case for ${\hat{p}}_{\text{m}}$ and *p*_cl_, m. Non-invasive estimation methods exist to backpropagate brachial pressures to central aortic pressure using transfer functions [18–20], but—owing to the large inter-individual variability and disease dependence of pressure wave augmentation—methods tend to produce errors of up to 40%, which are even beyond the range of ±10% probed in this study. However, in the CoA cases studied, even for *p*_op_, m, discrepancies of up to 56% were observed between invasive measurement and cuff estimation.

### (c) Influence of uncertainties on EM LV model output

While input data uncertainty had a major impact in fitting the Wk3 parameters, where a change of ±10% led to a variability in estimated parameters of up to +79% and −87%, respectively, these were not propagated forward to the haemodynamic output parameters used for measuring the goodness of fit of the global EM LV model. As shown in table 4 and illustrated in figure 4, the sensitivity to misfit in Wk3 parameters due to *p*_op_, m and *p*_cl_, m was ≪ 1. Misfit due to ${\hat{p}}_{\text{m}}$ was slightly more significant, but also always <1. Although the variation of *p*_cl,m_ has an equal influence on *Z* as ${\hat{p}}_{\text{m}}$ in the Wk3 model, at least for the AS cases, this influence is not observed in the EM simulation results, where only ${\hat{p}}_{\text{m}}$ had a major impact. To find a detailed explanation for this behaviour is a complex endeavour, as EM model outputs are influenced by multiple model parameters related to active stress, passive material properties as well as model geometry. Misfit obtained for the eight additional perturbed cases with combined input variability also remained less than 1 (see electronic supplementary material, table S5). As such, the haemodynamic output of the EM LV model is highly robust to the fitting errors the Wk3 parameters may be afflicted with, at least in the range ±10% probed.

## 5. Conclusion

This study reports on the development of an integrated workflow comprising anatomical modelling, clinical data pre-processing and parameter fitting, that is sufficiently automated to build a larger *virtual cohort* of 17 EM LV models. A global–local optimization method for identifying the parameters of a Wk3 model of afterload has been developed which is able to robustly and uniquely identify sets of Wk3 parameters. Results of Wk3 fitting demonstrated a significant dependence of parameters on input data uncertainty, suggesting that an accurate parametrization of a Wk3 afterload model may not be feasible in the absence of invasive pressure measurements. However, the haemodynamic output of the combined EM LV model remained virtually unaffected, indicating that organ-scale models may be able to replicate global haemodynamic behaviour of the LV fairly accurately, even when using non-invasively estimated pressure data that are afflicted with higher uncertainties.

## Supplementary Material

Data preprocessing, detailed methodolgy and additional results

## Data accessibility

For the input data see the electronic supplementary material.
