# Supplementary material for: Personalization of electro-mechanical models of the pressure-overloaded left ventricle: fitting of Windkessel-type afterload models
Source: Philos Trans A Math Phys Eng Sci. 2020 May 25;378(2173):20190342. doi: 10.1098/rsta.2019.0342 (PMC7287328; doi:10.1098/rsta.2019.0342)
Supplement: Data preprocessing, detailed methodolgy and additional results [file rsta20190342supp1.pdf]

PHILOSOPHICAL TRANSACTIONS A

rsta.royalsocietypublishing.org

Research

Article submitted to journal

Subject Areas:
biophysics, mathematical modelling, cardiology

Keywords:
aortic stenosis, coarctation, pressure gradient, heart failure

Author for correspondence:
Gernot Plank
e-mail: gernot.plank@medunigraz.at

Supplementary material for:

Personalization of electro-mechanical models of the pressure-overloaded left ventricle: Fitting of Windkessel-type afterload models

*Laura Marx[1], *Matthias A. F. Gsell[1], Armin Rund[2], Federica Caforio[1], Anton J. Prassl[1], Gabor Toth-Gayor[4], Titus Kuehne[3], Christoph M. Augustin[1] and Gernot Plank[1]

[1]Gottfried Schatz Research Center for Cell Signaling, Metabolism and Aging - Division of Biophysics, Medical University Graz, Austria
[2]Institute for Mathematics and Scientific Computing, University of Graz, Austria
[3]Dept. of Congenital Heart Diseases and Pediatric Cardiology, German Heart Institute Berlin, Germany
[4]Dept. of Cardiology, Medical University Graz, Austria
∗These authors contributed equally to the study.

© The Authors. Published by the Royal Society under the terms of the Creative Commons Attribution License http://creativecommons.org/licenses/by/4.0/, which permits unrestricted use, provided the original author and source are credited.

THE ROYAL SOCIETY
PUBLISHING

rsta.royalsocietypublishing.org Phil. Trans. R. Soc. A 0000000

1. Methods

(a) Data Pre-processing

In general, acquired clinical data are not suitable for being immediately interfaced with computational models, since data are not acquired synchronously at the same time. Rather, the time elapsed between acquisitions of the disparate data may be significant, in the range between minutes to days, and physiological conditions may be different, for instance, between acquisitions with and without sedation. These variabilities lead to inherent inconsistencies in the data which have to be considered in the process when identifying parameters of the underlying assumed model. Thus, careful pre-processing that factors in the specific circumstances of data acquisition is vital to obtain consistent input and validation data for modelling.

(i) Volumetric pre-processing

Information on LV volume, $V_{\rm lv}$, is obtained from the clinical data in two distinct ways. The static LV volume at a given instant during a cardiac cycle was derived from 3DWH MRI scans acquired during diastasis. Segmentation of the static 3DWH image stacks yields the most accurate measure of the LV blood pool volume for one given instant in time. On the other hand, the change of LV volume over time and thus the flow in and out of the LV were obtained from SAX cine MRI scans which were segmented using the semi-automatic clinical analysis software syngo.via (Siemens Healthcare GmbH, Germany) following the guidelines proposed in [1]. Although carried out consecutively within a short time window $<1\,\mathrm{hour}$, discrepancies between the volumetric information obtained from these sources inevitably arise. SAX cine MRI scans were acquired under breath-hold conditions which increases intra-thoracic pressure that compresses the heart and, thus, entails a notable reduction in LV blood pool volume. On the other hand, 3DWH scans were acquired using a navigator under physiological breathing conditions. These differences in acquisition conditions, combined with additional confounding factors such as the use of different segmentation strategies for cine and 3DWH or changes in heart rate between acquisitions, lead to a systematic mismatch in the volumetric data. Since anatomical models of a given patient are built on the basis of 3DWH scans, cine MRI based volume traces must be adjusted to reconcile the volume of the anatomical model with dynamic volumetric data. In this study the 3DWH volume data were considered as a reference since these were acquired under more physiological conditions. Discrepancies between cine MRI and 3DWH MRI due to temporal misalignment as a consequence of altered heart rate were corrected by selecting a matching relative instant within a cardiac cycle based on

$$\mathrm{TT_{cine}} = \mathrm{TT_{3DWH}} \cdot \frac{\mathrm{T_{cine}}}{\mathrm{T_{3DWH}}}, \tag{1.1}$$

where $\mathrm{TT_{cine}}$ and $\mathrm{TT_{3DWH}}$ denote the MRI trigger times and $\mathrm{T_{cine}}$ and $\mathrm{T_{3DWH}}$ the related cycle length of the analyzed heart beat. The difference between LV volumes obtained from the 3DWH segmentations ($\mathrm{V_{3DWH}}$) and from cine MRI corresponding to the cine trigger time ($\mathrm{V_{TTcine}}$), is equivalent to the value of the horizontal shift ΔV of the volume curve (Eq. 1.2, see Fig. 1a). Thus, assuming that the dynamics of the cine MRI-based volume traces is accurate, but shifted by an offset relative to $\mathrm{T_{3DWH}}$, volume traces can be reconciled by subtracting the offset

$$\Delta V = \mathrm{V_{3DWH}} - \mathrm{V_{TTcine}}. \tag{1.2}$$

For the sake of parameterizing the afterload model, the flow q across the aortic valve – which is $q = -\frac{\mathrm{d}V}{\mathrm{d}t}$ during ejection and $q = 0$ else (see Fig. 1a) – is the variable of primary interest, and not $V_{\rm lv}$ *per se*. However, the rather poor temporal resolution of acquired volume traces – with SAX MRI approx. $30\,\mathrm{ms}$ to $40\,\mathrm{ms}$ are feasible – combined with additional jitter introduced by the segmentation procedures leads to oscillatory flow curves $q(t)$ due to differentiation. These issues were addressed by smoothing the raw volume traces using cubic splines. Volume traces were split into two parts and interpolated from both ends towards each other. Polynomials of the third degree were used to enforce the derivative to meet $\frac{\mathrm{d}V}{\mathrm{d}t} = 0$ at the onset of ejection, at end-systole and end-diastole.

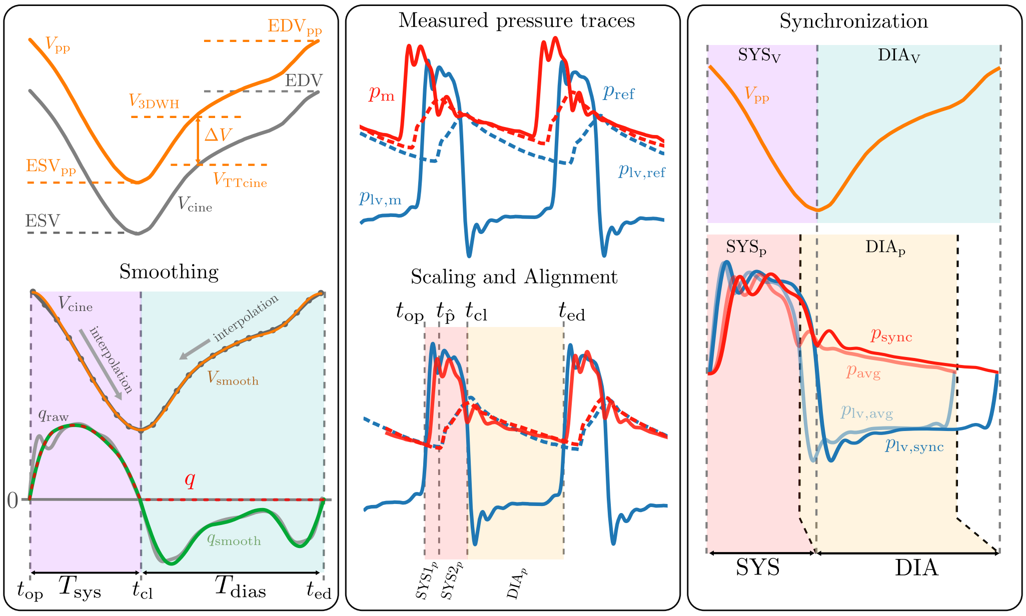

(a) Volumetric pre-processing (b) Pressure pre-processing (c) p-V-Synchronization

Figure 1: Schematic figure showing preprocessing steps including volume preprocessing to adjust cine-MRI traces to 3DWH MRI and smoothing (a), pressure preprocessing to find an average pressure beat (b) and p-V-Synchronization to adapt pressure traces to the HR of volume traces (c).

(ii) Pressure pre-processing

Pressure traces are recorded using a pig tail catheter measuring several pressure beats in the left ventricle $p_{\mathrm{lv,m}}$ (solid blue line in Fig. 1b) as well as at the entrance port, arteria femoralis $p_{\mathrm{lv,ref}}$ (dashed blue line), which is used as a reference. Consecutively, the catheter is pulled out of the ventricle through the aortic valve and pressure trace in aorta ascendens $p_{\mathrm{ao,m}}$ – shown in solid red together with the reference (dashed red) – is recorded. For the catheterisation procedure patient is under anaesthesia, whereas during echocardiography patient is in an awake state, which may lead to diverging measurement results (e.g. due to different HR). Thus, combining the outcome of both procedures for fitting should be avoided. Therefore, the pressure drop $\Delta p_{\mathrm{av,m}}$ across the aortic valve was calculated as the peak-to-peak difference of $p_{\mathrm{lv,m}}$ and p_{m} for CoA cases, whereas for AS cases results from echocardiography were used in combination with cuff measurements.

Recordings from catheterisation need to be pre-processed as during this highly invasive procedure systemic pressure may rise (visible as horizontal shift in reference traces) and HR may change. Further, even though measurements are ECG-triggered, individual traces do not start at the exact same instant of time within the cardiac cycle, making a time alignment necessary. Pressure traces contain several beats and the goal is to get a single representative averaged LV and aortic pressure beat (see Fig. 1b).

First the change in systemic pressure was corrected by finding the average minimum for all beats in both reference traces and by shifting p_{m} and its reference p_{ref} by the difference so that the two reference traces share the same baseline (see Fig. 1b).

To shift and scale the curves on the time axis some distinct points within the individual traces have to be identified, namely the starting point of the beats (aortic valve opening pressure $p_{\mathrm{op,m}}$), the peak values $\hat{p}_{\mathrm{m}}$ (approximately minimum of p_{ref}), the closing pressure of the valve $p_{\mathrm{cl,m}}$ and the end of the beat (equivalent to the start of the next beat). By simply looking for the starting and end point of a beat and scaling the beat as a whole, the resulting curve would not be representative for other heart rates. With decreasing heart rate only very small changes in duration of the ejection

occur, whereas a significant difference is seen in the duration of diastole [2].

Further, the assumption was made that minima of p_{m} beats are equivalent to the diastolic pressure and to $p_{\mathrm{op,m}}$. The time points of $p_{\mathrm{op,m}}$ in p_{m} corresponding to the value in $p_{\mathrm{lv,m}}$ are used as starting points for the individual beats.

To find the dicrotic notch in p_{m}, which equals the closing pressure of the aortic valve $p_{\mathrm{cl,m}}$, the time index of the peak reference pressure is used, followed by searching for a pressure minimum within a certain time frame (about $50\,\mathrm{ms}$, window size may be varied depending on the shape of the dicrotic notch). The resulting $p_{\mathrm{cl,m}}$ values need to be found in $p_{\mathrm{lv,m}}$ to retrieve the corresponding time indices for valve closing. In this way, three time intervals (SYS1, SYS2 and DIA) which have to be aligned and scaled separately can be found to obtain $p_{\mathrm{ao,avg}}$ and $p_{\mathrm{lv,avg}}$ (see Fig. 1b and Fig. 1c). The first two intervals make up the ejection phase (SYS) and the last interval corresponds to diastole (filling phase) of the cardiac cycle.

Additionally, RC-time was estimated with the decay time method [3] and solved via an optimization approach using the relation $p(t) = \alpha\, e^{-\frac{t}{\tau}}$ for the decay of pressure during diastole ($\tau = RC$). As from measured $p_{\mathrm{ao,m}}$ the time t_{ed} and pressure p_{ed} at the end of diastole is known $p(t_{\mathrm{ed}}) = p_{\mathrm{ed}} = \alpha e^{-\frac{t_{\mathrm{ed}}}{\tau}}$ and therefore α can be written as $\alpha = p_{\mathrm{ed}} e^{\frac{t_{\mathrm{ed}}}{\tau}}$ so that $p(t) = p_{\mathrm{ed}} e^{\frac{t_{\mathrm{ed}}-t}{\tau}}$. The unknown τ is optimized according to $\min_{\tau} \|p - p_{\mathrm{m}}\|^2_{[t_0, t_{\mathrm{ed}}]}$ where t_0 is the time point t_{cl} of aortic valve closing plus 10% of the total cycle length T_{cyc}.

(iii) Pressure-volume synchronization

The averaged pressure beats p_{avg} and $p_{\mathrm{lv,avg}}$ were synchronized to match the cycle length of the volume traces. This was again done separately for the ejection phase (SYS) and the diastolic phase (DIA) visualized in Fig. 1c.

(b) Empirical Reference Data

To further corroborate physiological box constraints in AS cases for which no invasive data were recorded in the *CARDIOPROOF* study [4], a statistical analysis was conducted on a reference group of $N = 290$ patients treated for AS. All patients in the reference group had moderate to severe AS, undergoing invasive hemodynamic assessment to decide about the necessity of surgical or percutaneous treatment of the valve disease. During this procedure ventricular and aortic pressure are measured continuously with a $1.8\,\mathrm{mm}$ double-lumen fluid filled catheter in a resting condition in order to determine the transvalvular pressure gradient, $\Delta p_{\mathrm{av,m}}$. The derived empirical values of the cohort (co) are summarized in Tab. 1 and are used as reference values for scaling.

Table 1: Mean and standard deviation of empirical values obtained from evaluation of $N = 290$ pressure traces. Opening pressure $p_{\mathrm{op,co}}$ of the aortic valve, closing pressure $p_{\mathrm{cl,co}}$ and peak pressure in the aorta $\hat{p}_{\mathrm{co}}$ are listed. $\mathrm{MAP}_{\mathrm{AUC,co}}$ is the mean arterial pressure estimated by the area under the curve (AUC) and $RC_{\mathrm{l2,co}}$ is the diastolic decay time estimated by the decay time method in combination with an optimization approach using the l2-norm.

Age<br>years	$p_{\mathrm{op,co}}$<br>kPa	$p_{\mathrm{cl,co}}$<br>kPa	$\hat{p}_{\mathrm{co}}$<br>kPa	$\mathrm{MAP}_{\mathrm{AUC,co}}$<br>kPa	$RC_{\mathrm{l2,co}}$<br>ms
77.94 (9.73)	8.31 (1.65)	13.77 (2.22)	16.27 (2.57)	11.03 (1.79)	919.97 (220.24)

(c) Afterload Model

Values of ω_i in Manuscript Eq. 2.3 are computed as follows:

$$\omega_0 = \frac{\gamma_0}{(\hat{p}_{\mathrm{co}})^2}, \qquad \omega_1 = \frac{\gamma_1}{(p_{\mathrm{op,co}})^2}, \qquad \omega_2 = \frac{\gamma_2}{(p_{\mathrm{cl,co}})^2}, \tag{1.3}$$

where $\hat{p}_{\mathrm{co}}$, $p_{\mathrm{op,co}}$ and $p_{\mathrm{cl,co}}$ result from the respective mean values of the cohort data (see Tab. 1) and the weighting factors γ_i were chosen so that each term of the cost functional contributes to the same extent to the overall cost ($\gamma_0 = \gamma 1 = 100.0$, $\gamma_2 = 10.0$).

The bounds b_i used in Manuscript Eq. 2.4 were chosen to lie within physiologically realistic ranges and are derived from the cohort data (see Eq. 1.4). Their values are

$$\begin{aligned} b_0 &= \left(\frac{RC}{T_{\mathrm{dia}}}\right)_{\mathrm{co,min}} = 1.1980, \\ b_1 &= \left(\frac{RC}{T_{\mathrm{dia}}}\right)_{\mathrm{co,max}} = 5.1819, \\ b_2 &= \left(\frac{p_{\mathrm{cl}}}{\mathrm{MAP}}\right)_{\mathrm{co,q10}} = 1.1232, \\ b_3 &= \left(\frac{p_{\mathrm{cl}}}{p_{\mathrm{op}}}\right)_{\mathrm{co,q10}} \cdot p_{\mathrm{op,m}} = 1.3714 \cdot p_{\mathrm{op,m}}. \end{aligned} \tag{1.4}$$

(d) Electromechanical Model

(i) Electrophysiology of the LV

A reaction-eikonal (R-E) model [5] was employed to generate electrical activation sequences which serve as a trigger for active stress generation in cardiac tissue. The hybrid R-E model combines a standard reaction-diffusion (R-D) model based on the monodomain equation with an eikonal model. Briefly, the eikonal equation is given as

$$\begin{cases} \sqrt{\nabla_{\mathbf{X}} t_{\mathrm{a}}^{\top}\, \mathbf{V}\, \nabla_{\mathbf{X}} t_{\mathrm{a}}} &= 1 \quad \text{in } \Omega_0, \\ t_{\mathrm{a}} &= t_0 \quad \text{on } \Gamma_0^*, \end{cases} \tag{1.5}$$

where $(\nabla_{\mathbf{X}})$ is the gradient with respect to the end-diastolic reference configuration Ω_0; t_{a} is a positive function describing the wavefront arrival time at location $\mathbf{X} \in \Omega_0$; and t_0 are initial activations at locations $\Gamma_0^* \subseteq \Gamma_0$ with Γ_0 the boundary of Ω_0. The symmetric positive definite 3×3 tensor $\mathbf{V}(\mathbf{X})$ holds the squared velocities $(v_{\mathrm{f}}(\mathbf{X}), v_{\mathrm{s}}(\mathbf{X}), v_{\mathrm{n}}(\mathbf{X}))$ associated to the tissue eigenaxes, referred to as fibre $(\mathbf{f}_0)$, sheet $(\mathbf{s}_0)$, and sheet normal $(\mathbf{n}_0)$ orientations. The arrival time function $t_{\mathrm{a}}(\mathbf{X})$ was subsequently used in a modified monodomain R-D model given as

$$\beta C_{\mathrm{m}} \frac{\partial V_{\mathrm{m}}}{\partial t} = \nabla_{\mathbf{X}} \cdot \sigma_{\mathrm{i}} \nabla_{\mathbf{X}} V_{\mathrm{m}} + I_{\mathrm{foot}} - \beta I_{\mathrm{ion}} \quad \text{in } \Omega_0, \tag{1.6}$$

where an arrival time dependent foot current, $I_{\mathrm{foot}}(t_{\mathrm{a}})$, was added, which is designed to mimic subthreshold electrotonic currents to produce a physiological foot of the action potential. The key advantage of the R-E model is its ability to compute activation sequences at much coarser spatial resolutions that are not afflicted by the spatial undersampling artefacts leading to conduction slowing or even numerical conduction block, as it is observed in standard R-D models. Ventricular EP was represented by the Tusscher–Noble–Noble–Panfilov model of the human ventricular myocyte [6]. Note that activation sequences and electrical source distribution in the LV (1.5, 1.6) were computed in its end-diastolic configuration Ω_0, that is, any effects of deformation upon electrotonic currents remained unaccounted for.

(ii) Active and passive mechanics in the LV and aorta

The deformation of the heart is governed by imposed external loads such as pressure in the cavities or from surrounding tissue and active stresses intrinsically generated during contraction.

Tissue properties of the LV myocardium and the aorta are characterized as a hyperelastic, nearly incompressible, anisotropic material with a non-linear stress-strain relationship. Mechanical deformation was described by Cauchy's equation of motion under stationary equilibrium assumptions leading to a quasi-static boundary value problem

$$-\nabla_{\mathbf{X}} \cdot \mathbf{F}\mathbf{S}(\mathbf{u}, t) = \mathbf{0} \quad \text{in } \Omega_0, \tag{1.7}$$

for $t \in [0, T]$, where $\mathbf{u}$ is the unknown displacement, $\mathbf{F}$ is the deformation gradient, $\mathbf{S}$ is the second Piola–Kirchhoff stress tensor, and $(\nabla_{\mathbf{X}}\, \cdot)$ denotes the divergence operator in the Lagrange reference configuration.

The total stress $\mathbf{S}$ was additively decomposed according to

$$\mathbf{S} = \mathbf{S}_{\text{pas}} + \mathbf{S}_{\text{act}}, \tag{1.8}$$

where $\mathbf{S}_{\text{pas}}$ and $\mathbf{S}_{\text{act}}$ refer to the passive and active stresses, respectively. Passive stresses were modelled based on the constitutive equation

$$\mathbf{S}_{\text{pas}} = 2\frac{\partial \Psi(\mathbf{C})}{\partial \mathbf{C}} \tag{1.9}$$

given a hyper-elastic and transversely isotropic strain-energy function Ψ

$$\Psi_{\text{Guc}}(\mathbf{C}) = \frac{\kappa}{2}(\log J)^2 + \frac{C_{\text{Guc}}}{2}\left[\exp(\mathcal{Q}) - 1\right] \tag{1.10}$$

by Guccione et al. [7]. Here, the term in the exponent is

$$\begin{aligned}\mathcal{Q} = {} & b_{\text{f}}(\mathbf{f}_0 \cdot \overline{\mathbf{E}}\mathbf{f}_0)^2 + b_{\text{t}}\left[(\mathbf{s}_0 \cdot \overline{\mathbf{E}}\mathbf{s}_0)^2 + (\mathbf{n}_0 \cdot \overline{\mathbf{E}}\mathbf{n}_0)^2 + 2(\mathbf{s}_0 \cdot \overline{\mathbf{E}}\mathbf{n}_0)^2\right] + \\ & 2b_{\text{fs}}\left[(\mathbf{f}_0 \cdot \overline{\mathbf{E}}\mathbf{s}_0)^2 + (\mathbf{f}_0 \cdot \overline{\mathbf{E}}\mathbf{n}_0)^2\right]\end{aligned} \tag{1.11}$$

and $\overline{\mathbf{E}} = \frac{1}{2}(\overline{\mathbf{C}} - \mathbf{I})$ is the modified isochoric Green–Lagrange strain tensor, where $\overline{\mathbf{C}} := J^{-2/3}\mathbf{C}$ with $J = \det \mathbf{F}$. Default values of $b_{\text{f}} = 18.48$, $b_{\text{t}} = 3.58$, and $b_{\text{fs}} = 1.627$ were used. The parameter C_{Guc} was used to fit the LV model to an empirical Klotz relation [8] by a combined unloading and re-inflation procedure. In the aorta, unlike in previous studies [9], we refrained from assigning fibre structures, since our efforts were primarily focused on modelling the biomechanics of the LV and, to a lesser degree, the aorta.

Thus, in absence of information on structural anisotropy, an isotropic neo-Hookean model [10] was used

$$\Psi_{\text{Neo}}(\mathbf{C}) := \frac{\kappa}{2}(J - 1)^2 + \frac{C_{\text{Neo}}}{2}\left(\text{tr}(\overline{\mathbf{C}}) - 3\right). \tag{1.12}$$

The bulk modulus κ, which serves as a penalty parameter to enforce nearly incompressible material behaviour, was chosen as $\kappa = 650\,\text{kPa}$ in both (1.10) and (1.12).

A simplified phenomenological contractile model was used to represent active stress generation [11]. Owing to its small number of parameters and its direct relation to clinically measurable quantities such as peak pressure and the maximum rate of rise of pressure, this model is fairly easy to fit and thus very suitable for being used in clinical EM modelling studies. Briefly, the active stress transient is given by

$$S_{\text{a}}(t, \lambda) = \hat{S}_{\text{a}}\, \phi(\lambda)\, \tanh^2\left(\frac{t_{\text{s}}}{\tau_{\text{C}}}\right) \tanh^2\left(\frac{t_{\text{dur}} - t_{\text{s}}}{\tau_{\text{R}}}\right), \qquad \text{for } 0 < t_{\text{s}} < t_{\text{dur}}, \tag{1.13}$$

where $\hat{S}_{\text{a}}$ is the peak isometric tension, $\phi(\lambda)$ is a nonlinear function dependent on fibre stretch $\lambda = |\mathbf{F}\mathbf{f}_0|$ describing the length dependence of active stress generation, t_s is the onset of contraction, τ_C is the upstroke time constant, t_{dur} is the active stress transient duration and τ_R is the downstroke time constant.

The active stress tensor in the reference configuration Ω_0 induced in fibre direction $\mathbf{f}_0$ is defined as

$$\mathbf{S}_{\text{a}} = S_{\text{a}}\,(\mathbf{f}_0 \cdot \mathbf{C}\mathbf{f}_0)^{-1}\,\mathbf{f}_0 \otimes \mathbf{f}_0, \tag{1.14}$$

with S_a defined in (1.13).

(iii) Mechanics boundary conditions at the LV and aorta

The different BCs applied to the LV models are summarized in Fig. 2. The springs attached to the aortic rim and at the pericardium are shown in Fig. 2A, as well as the pressure BC in the cavity at the endocardial surface. The pericardial springs penalize displacement in normal direction only and are gradually scaled from the apex to the base. Therefore, the distance in apico-basal direction was used to create a penalty map, see Fig. 2A. To avoid non-physiological rotation, further springs were attached to the septum, see Fig. 2B. The location of the septal springs was selected automatically by constructing a local coordinate system spanned by the centers of the apical region, the Mitral valve (MV) and the Aortic valve (AV).

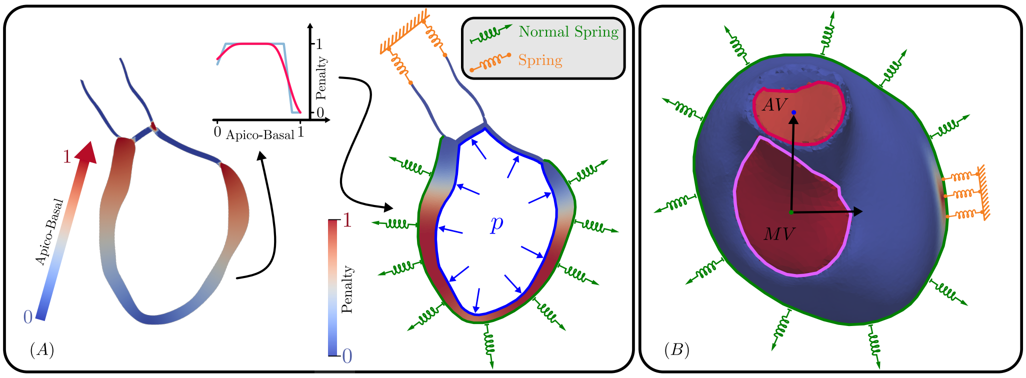

Figure 2: Boundary conditions applied to the LV models.

(e) Optimization

The optimization problem derived in Manuscript Sec. 2(b)i is non-convex and, hence, may possess several local minima. Thus, when solving this problem using an automated approach caution is warranted as the optimization may terminate with a non-competitive minimum. To circumvent this problem a combined global-local optimization method was applied. First, a neighborhood of a competitive local minimum was identified by stochastic sampling, which was then further refined by local optimization using adjoint-based derivatives.

Specifically, stochastic Sobol sampling [12,13] was implemented to generate 5000 different parameter sets for Z, R and C within a sufficiently large box. The cost for each set was evaluated, the set of lowest cost determined and the box re-centered around this candidate set, see Fig. 3A. The procedure was iteratively repeated until the candidate set was found to lie within the center of the box, measured as 80% of the box size. The box centers are illustrated by dotted lines in Fig. 3A. Shrinking steps were applied then by incrementally narrowing down the search range for each parameter to better resolve the topography of the cost functional, see Fig. 3B.

The optimal result found from stochastic sampling was used then as an initial guess for local optimization. Here, quasi-Newton methods based on adjoint-based exact discrete derivatives were applied.

2. Results

(a) Data Pre-processing

For $N_{\mathrm{CoA}} = 7$ cases, aortic pressure and volume traces were pre-processed according to Sec. 1(a)i–iii in this supplement. Aortic valve opening pressure $p_{\mathrm{op,m}}$, peak pressure $\hat{p}_{\mathrm{m}}$ and valve closing pressure $p_{\mathrm{cl,m}}$ were extracted from the processed pressure trace p_{m}. End-diastolic LV pressure $p_{\mathrm{lv,ed}}$ was obtained from the processed LV pressure trace $p_{\mathrm{lv,m}}$.

Due to the absence of measured pressure traces, only volume pre-processing, see Sec. 1(a)i, was applied to the $N_{\mathrm{AS}} = 10$ AS cases. Aortic valve opening pressure $p_{\mathrm{op,m}}$ and peak pressure

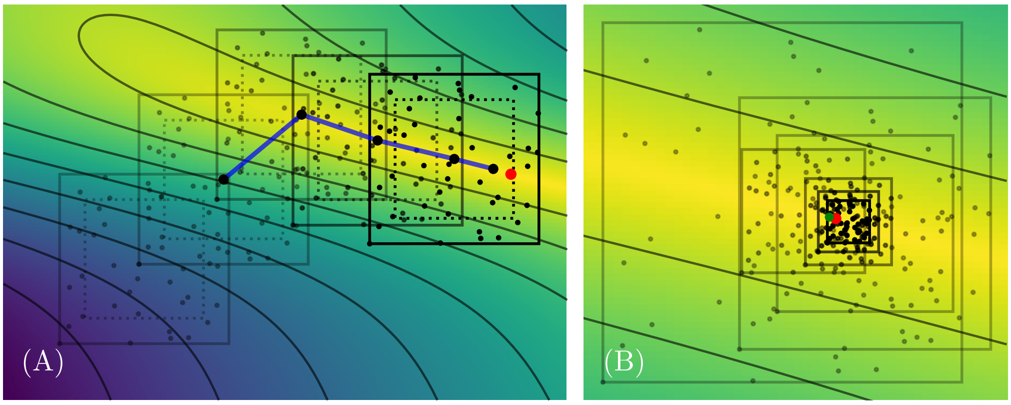

Figure 3: Global optimization algorithm.

$\hat{p}_{\mathrm{m}}$ were estimated as $p_{\mathrm{dia,cuff}}$ and $p_{\mathrm{sys,cuff}}$, respectively. Valve closing pressure was estimated as

$$p_{\mathrm{cl,m}} = p_{\mathrm{op,m}} + \bar{\delta} p_{\mathrm{co}} \left(\hat{p}_{\mathrm{m}} - p_{\mathrm{op,m}}\right), \tag{2.1}$$

where $\bar{\delta} p_{\mathrm{co}}$ was computed as the mean value of

$$\delta p_{\mathrm{co}} = \frac{p_{\mathrm{cl,co}} - p_{\mathrm{op,co}}}{\hat{p}_{\mathrm{co}} - p_{\mathrm{op,co}}} \tag{2.2}$$

based on empirical reference data, see Sec. 1(b). $p_{\mathrm{lv,ed}}$ for AS cases was obtained from the empirical reference data. Pre-processed pressure and volume values for all cases are summarized in Tab. 2 and serve as input for the Wk3 model fitting.

Pressure pre-processing of case 01-CoA and 04-CoA resulted in a negative Δp_{av} also yielding negative values for Z_{av}. Due to physiological reasons $Z_{\mathrm{av}} = 0.0$ was used as input for EM LV simulations for the respective cases.

(b) Fitting of the Afterload Model

Afterload fitting results of all cases are summarized in Tab. 3.

(c) EM LV Model Parameterization

(i) Anatomical modelling

Patient-specific anatomical models of all cases (N=17) were generated following established workflows mentioned previously and are visualized in Fig 4.

(ii) Passive biomechanics

Constitutive relations are represented in terms of the Guccione Model (see Eq. 1.10). From the anatomical models at ED state, a stress-free reference configuration is computed using default material parameters and $p_{\mathrm{lv,ed}}$ in the LV (for AS cases the empirical value of 2.8 kPa is used, while for CoA cases patient-specific values extracted from invasive pressure measurements are taken). This is accomplished by unloading the geometry using a backward displacement method [14]. The EDPVR is fitted to the empiric Klotz EDPVR using default values for parameters $b_{\mathrm{f}} = 18.48$, $b_{\mathrm{t}} = 3.58$ and $b_{\mathrm{fs}} = 1.627$ from literature [7] adapting only the stiffness parameter C_{Guc}. Values for the fitted passive material parameter C_{Guc} are shown in Tab. 4 for all cases.

Table 2: Pre-processed pre-treatment AS and CoA patient characteristics from SAX cine MRI, invasive catheterization, non-invasive cuff pressure recordings and echo-US including Sex, Age (y), heart rate (HR), end-diastolic volume (EDV), end-systolic volume (ESV), peak flow ($\hat{q}$), catheter pressures ($p_{\mathrm{op,m}}$, $p_{\mathrm{cl,m}}$ and $\hat{p}_{\mathrm{m}}$) and the peak to peak pressure drop across the valve ($\Delta p_{\mathrm{av,m}}$) for CoA patient cases, diastolic and systolic cuff pressures ($p_{\mathrm{dia,cuff}}$ and $p_{\mathrm{sys,cuff}}$) and estimated closing pressure ($p_{\mathrm{cl,m}}$) and pressure drop across aortic valve ($\Delta p_{\mathrm{av,m}}$) for AS patient cases. The impedance of the aortic valve Z_{v} is computed as $\Delta p_{\mathrm{av,m}}$ over $\hat{q}$.

Case ID	Sex	Age	HR	EDV	ESV	EF	$\hat{q}$	$p_{\mathrm{op,m}}$	$p_{\mathrm{cl,m}}$	$\hat{p}_{\mathrm{m}}$	$\Delta p_{\mathrm{av,m}}$	Z_{v}
		y	$\frac{1}{\mathrm{min}}$	ml	ml	%	$\frac{\mathrm{ml}}{\mathrm{ms}}$	kPa	kPa	kPa	kPa	kPa$\frac{\mathrm{ms}}{\mathrm{ml}}$
01-AS	M	15	69	239.98	112.17	53.26	0.59	6.53	12.53	15.20	0.90	1.52
02-AS	M	22	69	275.33	144.16	47.64	0.47	10.67	15.38	17.47	6.40	13.63
03-AS	M	62	70	220.46	129.45	41.28	0.35	10.67	15.28	17.33	9.87	27.94
04-AS	F	79	62	97.27	36.43	62.54	0.28	9.33	16.62	19.87	10.67	38.39
05-AS	M	54	78	175.86	59.26	66.30	0.53	9.47	15.93	18.80	10.53	19.72
06-AS	M	58	71	237.81	159.80	32.80	0.29	10.53	16.53	19.20	8.87	27.00
07-AS	F	66	68	120.34	44.57	62.96	0.30	11.47	16.82	19.20	9.73	32.32
08-AS	M	51	72	281.70	158.26	43.82	0.51	8.93	14.47	16.93	11.47	22.47
09-AS	F	62	55	128.28	38.68	69.85	0.30	9.87	14.67	16.80	12.67	42.86
10-AS	M	72	84	208.86	112.49	46.14	0.39	10.00	15.45	17.87	8.27	21.26
01-CoA	M	57	91	155.67	55.62	64.27	0.54	7.43	10.46	16.29	−3.28	−0.82
02-CoA	M	18	49	230.36	114.72	50.20	0.43	6.54	8.38	13.34	5.11	1.60
03-CoA	F	9	65	96.28	50.32	47.73	0.21	8.84	12.89	15.75	2.86	1.86
04-CoA	M	10	95	92.70	25.18	72.84	0.41	10.20	13.73	18.16	−6.77	−2.20
05-CoA	M	15	92	152.85	60.83	60.20	0.59	9.80	12.62	17.01	5.48	1.23
06-CoA	M	18	86	159.51	59.53	62.68	0.55	12.58	15.06	18.85	10.68	2.59
07-CoA	M	11	70	123.92	68.58	44.66	0.26	7.69	9.13	13.02	11.84	6.17

Table 3: Results of Wk3 fitting procedure for all cases.

Case ID	p_{op}	p_{cl}	$\hat{p}$	Z	R	C	RC
	kPa	kPa	kPa	kPa$\frac{\mathrm{ms}}{\mathrm{ml}}$	kPa$\frac{\mathrm{ms}}{\mathrm{ml}}$	$\frac{\mathrm{ml}}{\mathrm{kPa}}$	ms
01-AS	6.53	12.53	15.20	7.04	64.47	13.85	893.08
02-AS	10.67	15.38	17.47	6.08	86.87	16.01	1390.93
03-AS	10.67	15.29	17.33	7.71	125.38	10.34	1296.78
04-AS	9.33	16.62	19.87	19.12	206.16	5.10	1051.43
05-AS	9.47	15.93	18.80	7.28	89.32	10.33	922.79
06-AS	10.53	16.54	19.20	12.87	150.69	7.30	1100.48
07-AS	11.47	16.82	19.20	11.67	164.43	8.73	1435.28
08-AS	8.93	14.46	16.93	6.85	78.06	12.88	1004.98
09-AS	9.87	14.67	16.80	10.53	150.98	11.47	1731.80
10-AS	10.00	15.45	17.87	9.26	97.46	8.42	820.60
01-CoA	7.43	10.47	16.28	13.10	59.90	18.75	1123.08
02-CoA	6.54	8.52	13.34	12.75	80.40	38.66	3108.14
03-CoA	8.84	12.89	15.74	21.35	225.28	7.18	1617.87
04-CoA	10.20	13.80	18.16	14.07	113.61	11.50	1306.87
05-CoA	9.80	12.80	17.01	9.12	80.17	19.10	1531.09
06-CoA	12.58	15.55	18.85	8.44	103.94	19.85	2063.62
07-CoA	7.69	9.45	13.02	16.38	132.65	19.52	2588.77

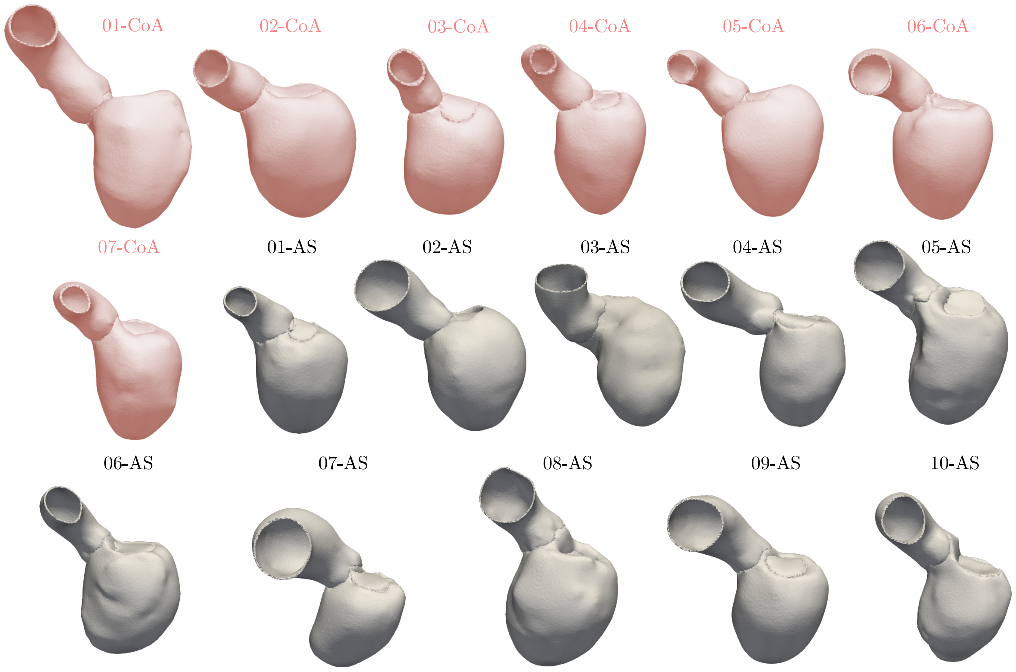

Figure 4: Patient-specific anatomical models of LV and aorta of all 17 cases generated from 3DWH MRI images. Models from patients suffering from CoA are visualized in red, whereas AS cases are shown in grey.

(iii) Active stress model

The active stress model was fitted using default values of $\{\tau_\mathrm{C} = 40\,\mathrm{ms},\ \hat{S}_\mathrm{a} = 100\,\mathrm{kPa},\ \tau_\mathrm{R} = 110\,\mathrm{ms}\}$ as initial guess. T_dur was initialized with the RT interval observed in the ECG. A linear mapping was used to correct the active stress model parameters. In the AS cases where measured pressure traces $p_\mathrm{lv,m}(t)$ were not available, only $\hat{S}_\mathrm{a}$ was iteratively adjusted by a fixed-point iteration $\hat{S}_{\mathrm{a},i+1} = \hat{S}_{\mathrm{a},i} \cdot \hat{p}_\mathrm{lv}/\hat{p}_{\mathrm{lv},i}$. Rate of rise τ_C and decline τ_R in the active stress model were adjusted accordingly using the shape of the volume trace $V_\mathrm{lv,m}$. For the CoA cases, $\frac{\mathrm{d}p_\mathrm{lv,m}}{\mathrm{d}t}|_\mathrm{max}$ and $\frac{\mathrm{d}p_\mathrm{lv,m}}{\mathrm{d}t}|_\mathrm{min}$ were used additionally to fit τ_C and τ_R.

As the focus of this study was on modelling systole, the diastolic isovolumetric relaxation and filling phases were not fitted. Fitted Parameters along with goodness of fit are summarized in Tab. 4 for all cases.

(d) Sensitivity Analysis

Sensitivity of fitting $\{Z, R, C\}$ to uncertainty in input data $\{p_\mathrm{op,m}, \hat{p}_\mathrm{m}, p_\mathrm{cl,m}\}$ was analyzed for all $N = 17$ cases by independently varying input variables within a $\pm 10\%$ range. Relative sensitivities averaged pathology specific are summarized in Tab. 5. Further, the relation between relative deviation ot the Wk3 parameters resulting from variation in input data and the original input pressure ratio $\frac{p_\mathrm{cl,m} - p_\mathrm{op,m}}{\hat{p}_\mathrm{m} - p_\mathrm{op,m}}$ was studied, see Fig. 5.

The impact upon EM LV was studied by using all possible $N = 6$ combinations of the extreme Wk3 parameters found and quantifying the deviation in model predictions $\{\Delta\mathrm{SV}, \Delta\mathrm{SW}, \Delta t_{\hat{p}}\}$ relative to the reference EM simulations where the mean parameters were used (see Manuscript Tab. 4). Results from 8 further simulations with combined input parameters variations can be found in Tab. 6.

Table 4: Fitted model parameters and goodness of fit for EM simulations shown in terms of deviations between simulated and measured values for EDV, ESV, EF, $\hat{p}$ and duration of ejection T_{sys}.

	Fitted Parameters					Goodness of Fit				
Case ID	C_{Guc}	$\hat{S}_a$ kPa	T_{dur} ms	τ_{C} ms	τ_{R} ms	Δ_{EDV} ml	Δ_{ESV} ml	Δ_{EF} %	$\Delta_{\hat{\mathrm{p}}}$ kPa	$\Delta_{\mathrm{T_{sys}}}$ ms
01-AS	0.40	75	430	105	60	0.03	0.89	0.36	0.16	10.53
02-AS	0.40	83	525	80	90	0.02	9.52	3.94	0.14	5.53
03-AS	0.40	68	535	90	70	0.01	10.54	4.78	0.32	6.63
04-AS	0.35	63	610	100	70	0.24	12.77	13.07	0.14	2.51
05-AS	0.40	68	430	70	90	0.01	0.14	0.08	0.24	1.43
06-AS	0.40	84	490	60	85	0.01	10.03	4.22	0.09	5.57
07-AS	0.40	73	485	70	80	0.09	4.79	3.96	0.21	26.26
08-AS	0.50	70	460	70	70	0.00	5.06	1.80	0.62	0.36
09-AS	0.40	90	610	70	100	0.2	7.00	5.50	0.49	31.92
10-AS	0.40	95	495	70	70	0.40	10.39	5.07	0.25	2.81
01-CoA	0.40	53	525	60	100	0.02	4.03	2.58	0.37	10.49
02-CoA	0.80	57	575	105	90	0.08	0.40	0.16	0.32	25.51
03-CoA	0.25	85	530	80	80	0.00	0.86	0.89	0.71	5.56
04-CoA	0.20	58	620	60	105	0.01	8.01	8.64	0.56	21.87
05-CoA	0.50	65	470	100	70	0.00	2.35	1.54	0.56	2.75
06-CoA	0.30	72	500	90	80	0.06	4.97	3.13	0.16	15.95
07-CoA	0.40	75	480	65	70	0.00	6.29	5.08	0.31	3.39

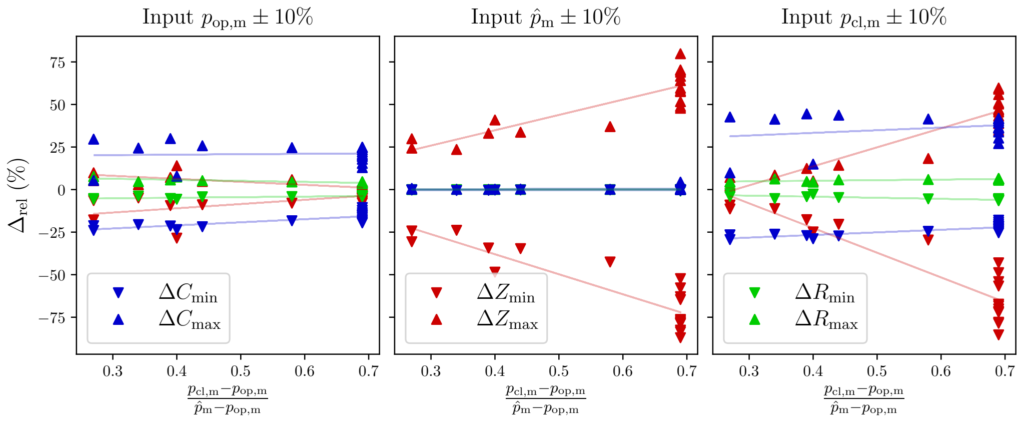

Figure 5: Scatter plot and trend lines of relative deviation of the Wk3 parameters of all $N = 17$ cases resulting from varied input data $p_{\mathrm{op,m}}$, $\hat{p}_{\mathrm{m}}$ and $p_{\mathrm{op,cl}}$ as a function of the original input pressure ratio.

3. Discussion

(a) Limitations of the Study

For AS cases no invasive pressure recordings were available. Thus, systolic LV and central aortic pressures needed as input for the parametrization of the Wk3 model relied on a simplistic estimation where central aortic pressure was assumed to be equal to brachial systolic pressure

Table 5: Max. ± relative deviation of Wk3 parameters for each pathology as a function of errors in the input data $p_{\mathrm{op,m}}$, $\hat{p}_{\mathrm{m}}$ and $p_{\mathrm{op,cl}}$.

Pathology		AS			CoA		
Input varied	Dev.	$Z_{\Delta\pm,\max}$ %	$R_{\Delta\pm,\max}$ %	$C_{\Delta\pm,\max}$ %	$Z_{\Delta\pm,\max}$ %	$R_{\Delta\pm,\max}$ %	$C_{\Delta\pm,\max}$ %
$p_{\mathrm{op,m}}$	±10%	$+2.27/-7.77$	$+4.62/-4.11$	$+24.92/-19.58$	$+14.07/-28.53$	$+7.67/-5.67$	$+29.97/-23.99$
$\hat{p}_{\mathrm{m}}$	±10%	$+79.82/-87.02$	$+0.18/-0.88$	$+4.39/-0.51$	$+40.89/-48.51$	$+0.12/-0.06$	$+0.70/-0.52$
$p_{\mathrm{cl,m}}$	±10%	$+59.67/-85.35$	$+6.51/-6.51$	$+41.88/-25.38$	$+18.25/-29.64$	$+6.24/-5.45$	$+41.88/-29.37$

Table 6: Relative deviation of results of EM-Simulations from initial fit for the cases 10-AS and 02-CoA using additional 8 varied Windkessel parameter sets as input, while keeping EM model parameters constant.

		Case 10-AS			Case 02-CoA		
Input varied	Deviation	SV ml	SW J	$t_{\hat{p}}$ ms	SV ml	SW J	$t_{\hat{p}}$ ms
	0%	110.77	2.58	345	115.31	1.44	280
		ΔSV %	ΔSW %	$\Delta t_{\hat{p}}$ %	ΔSV %	ΔSW %	$\Delta t_{\hat{p}}$ %
$p_{\mathrm{op,m}}, \hat{p}_{\mathrm{m}}, p_{\mathrm{cl,m}}$	+10%	−7.56	−6.31	0.87	−9.70	−6.76	2.50
$p_{\mathrm{op,m}}, \hat{p}_{\mathrm{m}}, p_{\mathrm{cl,m}}$	−10%	7.68	6.00	−1.45	9.67	5.44	−3.21
$\hat{p}_{\mathrm{m}}, p_{\mathrm{cl,m}}$	+10%	−7.00	−6.66	0.29	−8.09	−6.48	2.14
$\hat{p}_{\mathrm{m}}, p_{\mathrm{cl,m}}$	−10%	8.26	7.51	−1.74	8.58	5.99	−3.21
$p_{\mathrm{op,m}}, \hat{p}_{\mathrm{m}}$	+10%	−5.99	−4.37	−2.90	−8.47	−5.37	2.86
$p_{\mathrm{op,m}}, \hat{p}_{\mathrm{m}}$	−10%	7.83	4.30	4.93	7.14	2.51	−3.21
$p_{\mathrm{op,m}}, p_{\mathrm{cl,m}}/\hat{p}_{\mathrm{m}}$	+10%/−10%	7.85	6.81	9.28	3.50	1.46	−3.21
$p_{\mathrm{op,m}}, p_{\mathrm{cl,m}}/\hat{p}_{\mathrm{m}}$	−10%/+10%	−3.63	−3.33	−6.38	−3.27	−2.65	2.14

as measured by sphygmomanometry. It is well known that such estimates are highly inaccurate due to the large inter-individual variability in pressure wave augmentation [2,15].

While diastolic and mean arterial pressures are relatively constant throughout the arterial system and, thus, can be estimated from cuff measurements, this is not the case for systolic pressures. It is known that systolic pressure may be up to 40 mmHg (5.33 kPa) higher in the brachial artery than in the aorta [16]. This phenomenon of systolic pressure wave augmentation is attributed to an impedance mismatch between more compliant central vessels and the stiffer, less compliant more peripheral arterial vessels, which causes reflections of the forward travelling pressure wave. Central aortic pressure is therefore a composite of the forward travelling pressure wave and the reflected backward travelling wave, which combined augment pressure in the aorta relative to the brachial artery. How much the systolic pressure rises/diastolic pressure falls between aorta ascendens and the brachial artery is highly variable among patients depending on hemodynamic and pathophysiological characteristics such as body size and height, age, HR, stiffening of the aorta and general level of blood pressure [17]. This makes the extrapolation of pressure in the aorta ascendens from the sphygmomanometer measurements highly inaccurate. Beyond invasive measurements which may not always be viable non-invasive alternatives such as applanation tonometry exist, which may yield significantly more accurate estimates of systolic aortic blood pressure. For an overview of alternative non-invasive methods see, for instance, [18].

Beyond the significant measurement uncertainty, these wave transmission aspects are not taken into account by the Wk3 model. More elaborate Wk3 models that incorporate both reservoir- and wave-based aspects into a single lumped 0D model [19] or 1D models [20–24] may be able to better reflect vascular physiology, but have not been considered in this study. However, for providing an appropriate afterload to an EM model of the LV the use of a Wk3 model can be considered a fair trade-off. As shown in here, parameters can be identified fairly uniquely, the aortic pressure wave form approximates measurements sufficiently well and the physiological

details of the genesis of aortic pressure waveform is of lesser relevance for the questions being addressed with organ scale EM models.

References

1. Schulz-Menger J, Bluemke DA, Bremerich J, Flamm SD, Fogel MA, Friedrich MG, et al. Standardized image interpretation and post processing in cardiovascular magnetic resonance: Society for Cardiovascular Magnetic Resonance (SCMR) board of trustees task force on standardized post processing. J Cardiovasc Magn Reson. 2013;15(1):35.
2. Vlachopoulos C, O'Rourke M, Nichols WW. McDonald's blood flow in arteries: theoretical, experimental and clinical principles. CRC press; 2011.
3. Frank O. Die Grundform des arteriellen Pulses (The basic shape of the arterial pulse). Z Biol. 1899;37:483–526.
4. Fernandes JF, Goubergrits L, Brüning J, Hellmeier F, Nordmeyer S, da Silva TF, et al. Beyond pressure gradients: the effects of intervention on heart power in aortic coarctation. PloS one. 2017;12(1):e0168487.
5. Neic A, Campos FO, Prassl AJ, Niederer SA, Bishop MJ, Vigmond EJ, et al. Efficient computation of electrograms and ECGs in human whole heart simulations using a reaction-eikonal model. J Comput Phys. 2017;346:191–211.
6. Ten Tusscher KHWJ, Noble D, Noble PJ, Panfilov AV. A model for human ventricular tissue. Am J Physiol Heart Circ Physiol. 2004;286:H1573–H1589.
7. Guccione JM, Costa KD, McCulloch AD. Finite element stress analysis of left ventricular mechanics in the beating dog heart. J Biomech. 1995;28(10):1167 – 1177.
8. Klotz S, Dickstein ML, Burkhoff D. A computational method of prediction of the end-diastolic pressure-volume relationship by single beat. Nat Protoc. 2007;2(9):2152–8.
9. Augustin CM, Holzapfel GA, Steinbach O. Classical and all-floating FETI methods for the simulation of arterial tissues. Int J Numer Methods Eng. 2014;99(4):290–312.
10. Rivlin RS. Large Elastic Deformations of Isotropic Materials. I. Fundamental Concepts. Philosophical Transactions of the Royal Society A: Mathematical, Physical and Engineering Sciences. 1948;240(822):459–490.
11. Niederer SA, Plank G, Chinchapatnam P, Ginks M, Lamata P, Rhode KS, et al. Length-dependent tension in the failing heart and the efficacy of cardiac resynchronization therapy. Cardiovasc Res. 2011;89:336–343.
12. Joe S, Kuo FY. Constructing Sobol sequences with better two-dimensional projections. SIAM J Sci Comput. 2008;30(5):2635–2654.
13. Joe S, Kuo FY. Remark on Algorithm 659: Implementing Sobol's Quasirandom Sequence Generator. ACM Trans Math Softw. 2003;29(1):49–57.
14. Bols J, Degroote J, Trachet B, Verhegghe B, Segers P, Vierendeels J. A computational method to assess the in vivo stresses and unloaded configuration of patient-specific blood vessels. Journal of computational and Applied mathematics. 2013;246:10–17.
15. O'Rourke M, Blazek J, Morreels Jr C, Krovetz L. Pressure Wave Transmission along the Human Aorta: Changes with Age and in Arterial Disease. Circulation research. 1968;23(4):567–579.
16. Ohte N, Saeki T, Miyabe H, Sakata S, Mukai S, Hayano J, et al. Relationship between blood pressure obtained from the upper arm with a cuff-type sphygmomanometer and central blood pressure measured with a catheter-tipped micromanometer. Heart Vessels. 2007;22:410–415.
17. Muiesan ML, Salvetti M, Bertacchini F, Agabiti-Rosei C, Maruelli G, Colonetti E, et al. Central blood pressure assessment using 24-hour brachial pulse wave analysis. J Vasc Diagn Interv. 2014;2:141–148.
18. McEniery CM, Cockcroft JR, Roman MJ, Franklin SS, Wilkinson IB. Central blood pressure: current evidence and clinical importance. Eur Heart J. 2014;35:1719–1725.
19. Wang JJ, O'Brien AB, Shrive NG, Parker KH, Tyberg JV. Time-domain representation of ventricular-arterial coupling as a windkessel and wave system. Am J Physiol Heart Circ Physiol. 2003;284(4):H1358–H1368.
20. Formaggia L, Lamponi D, Quarteroni A. One-dimensional models for blood flow in arteries. J Eng Math. 2003;47(3-4):251–276.
21. Mynard JP, Nithiarasu P. A 1D arterial blood flow model incorporating ventricular pressure, aortic vaive ana regional coronary flow using the locally conservative Galerkin (LCG) method. Commun Numer Methods Eng. 2008;.
22. Alastruey J, Parker KH, Sherwin SJ. Arterial pulse wave haemodynamics. BHR Group - 11th International Conferences on Pressure Surges. 2012;p. 401–442.
23. Müller LO, Toro EF. A global multiscale mathematical model for the human circulation with emphasis on the venous system. Int J Numer Method Biomed Eng. 2014;30(7):681–725.

24. Blanco PJ, Watanabe SM, Dari EA, Passos MARF, Feijóo RA. Blood flow distribution in an anatomically detailed arterial network model: criteria and algorithms. Biomech Model Mechanobiol. 2014;13(6):1303–1330.
